# Intracellularly Localized PIN-FORMED8 Promotes Lateral Root Emergence in *Arabidopsis*

**DOI:** 10.3389/fpls.2019.01808

**Published:** 2020-01-31

**Authors:** Hyodong Lee, Anindya Ganguly, Richard Dongwook Lee, Minho Park, Hyung-Taeg Cho

**Affiliations:** Department of Biological Sciences, Seoul National University, Seoul, South Korea

**Keywords:** *Arabidopsis*, auxin, auxin transport, lateral root, PIN-FORMED8

## Abstract

PIN-FORMED (PIN) auxin efflux carriers with a long central hydrophilic loop (long PINs) have been implicated in organogenesis. However, the role of short hydrophilic loop PINs (short PINs) in organogenesis is largely unknown. In this study, we investigated the role of a short PIN, PIN8, in lateral root (LR) development in *Arabidopsis thaliana*. The loss-of-function mutation in *PIN8* significantly decreased LR density, mostly by affecting the emergence stage. *PIN8* showed a sporadic expression pattern along the root vascular cells in the phloem, where the PIN8 protein predominantly localized to intracellular compartments. During LR primordium development, *PIN8* was expressed at the late stage. Plasma membrane (PM)-localized long PINs suppressed LR formation when expressed in the *PIN8* domain. Conversely, an auxin influx carrier, AUX1, restored the wild-type (WT) LR density when expressed in the *PIN8* domain of the *pin8* mutant root. Moreover, LR emergence was considerably inhibited when AXR2-1, the dominant negative form of Aux/IAA7, compromised auxin signaling in the *PIN8* domain. Consistent with these observations, the expression of many genes implicated in late LR development was suppressed in the *pin8* mutant compared with the WT. Our results suggest that the intracellularly localized PIN8 affects LR development most likely by modulating intracellular auxin translocation. Thus, the function of PIN8 is distinctive from that of PM-localized long PINs, where they generate local auxin gradients for organogenesis by conducting cell-to-cell auxin reflux.

## Introduction

Auxin plays a critical role in plant growth and development by forming local concentration gradients. The formation of local auxin gradients is achieved by directional cell-to-cell auxin transport by auxin efflux and influx carriers. To date, three major families of auxin transporters have been identified: AUXIN-RESISTANT1 (AUX1)/AUX1-LIKEs (LAXs) for auxin influx and PIN-FORMEDs (PINs) and ATP-binding cassette Bs (ABCBs)/P-glycoproteins (PGPs) for auxin efflux. PIN proteins, unlike other families, generally play a critical role in establishing an auxin gradient because of their prominent asymmetric localization in the plasma membrane (PM), which enables directional flow of auxin from one cell to another. Additionally, environmental and developmental cues can dynamically alter auxin flows *via* PIN relocalization (Grunewald and Friml, 2010; Ganguly et al., 2012).

In *Arabidopsis thaliana*, the PIN family comprises eight members, and each PIN protein has 10 highly conserved transmembrane (TM) helices (five each at the N- and C-termini) and a central hydrophilic loop (HL) (Křeček et al., 2009; Ganguly et al., 2012). PINs are divided into two subgroups, depending on the HL length: long PINs including PIN1–PIN4, PIN6, and PIN7 that have a long HL of approximately 300 amino acid residues, and short PINs including PIN5 and PIN8 that have a much shorter HL ranging from 27–46 residues (Ganguly et al., 2010). Long PINs predominantly localize to the PM and exhibit distinct polarity to direct polar auxin flow. However, short PINs are localized in either intracellular compartments or the PM and are implicated in both intracellular translocation and cellular export of auxin (Mravec et al., 2009; Ganguly et al., 2010; Dal Bosco et al., 2012; Ding et al., 2012; Ganguly et al., 2014). In its native expression domain, PIN5 is expressed in the root and shoot vasculature and the cotyledon epidermis, where it is localized to the PM of pavement and guard cells in the cotyledon but to intracellular compartments in the root and shoot vasculature (Ganguly et al., 2014; Verna et al., 2015). Internally localized PIN5 seems to increase intracellular auxin levels and thus enhance auxin responses, as shown in the auxin-sensitive root hair system where root hair-specifically expressed PIN5 localizes to internal compartments and enhances root hair growth (Ganguly et al., 2010). PIN8 was shown to be expressed in the leaf vein and the pollen, where it is localized to intracellular compartments (Dal Bosco et al., 2012; Ding et al., 2012; Sawchuk et al., 2013; Verna et al., 2015). Conversely, when ectopically expressed, PIN8 shows intracellular or PM localization in the root epidermal cells, depending on the developmental stage (Ganguly et al., 2014). Whenever it is localized predominantly to the PM, PIN8, like other long PINs, shows an obvious auxin-exporting activity in the *Arabidopsis* root hair system and tobacco suspension cells (Ganguly et al., 2010). Therefore, it is conceivable that, depending on the cell type and developmental stage, short PINs are able to not only regulate intracellular auxin homeostasis but also facilitate intercellular auxin transport.

Polar auxin transport is intimately involved in the development of both primary and lateral roots (LRs) (Atkinson et al., 2014). LRs are the major determinants of the root system architecture and are critical for the acquisition of water and nutrients from the soil and anchorage of the plant body (Hochholdinger and Zimmermann, 2008; Dastidar et al., 2012). Auxins play a pivotal role in LR development at almost every stage, including priming, initiation, primordium development, and emergence [for a recent review, (Du and Scheres, 2018)]. In the basal meristem of the root, auxins seem to prime the pericycle cells contacting the xylem pole of the root vasculature into LR founder cells (LRFCs) (Parizot et al, 2008; Péret et al., 2009a; Péret et al., 2009b; Lavenus et al., 2013). LRFCs then undergo a series of anticlinal divisions to produce several initial daughter cells, which then undergo a coordinated process of anticlinal, periclinal, and tangential divisions to give rise to the LR primordium. The LR primordium then continues to grow through the cortical and epidermal cells of the root (Lucas et al., 2013; von Wangenheim et al., 2016).

Both auxin influx and efflux carriers play important roles in LR formation. The auxin influx carrier AUX1 facilitates auxin transport from the shoot vasculature to the root system for LR initiation (Marchant et al., 2002; De Smet et al., 2007). In addition, selective induction of the influx carrier LIKE-AUX3 (LAX3) in cortical and epidermal cells adjacent to an emerging LR primordium stimulates auxin-dependent cell-wall remodeling, which facilitates cell separation for LR emergence (Swarup et al., 2008; Péret et al., 2013). During LR formation, the auxin efflux carrier PIN3 localizes to the inner membrane of the root endodermal cells and is believed to facilitate auxin reflux into the LRFCs for the first asymmetric anticlinal division of founder cells (Marhavy et al., 2013; Péret et al., 2013). Cytokinin-mediated PIN1 degradation and re-localization from one cellular domain to another in the LR primordium has been shown to be important for LR emergence (Marhavy et al., 2011; Marhavy et al., 2014). Although loss-of-function mutations in several long PINs have been shown to disrupt LR formation and alter the root branching pattern (Du and Scheres, 2018), the role of short PINs in LR development has not yet been investigated.

While PM-localized long PINs affect organogenesis by forming local auxin gradients resulting from auxin reflux by their cell-to-cell auxin-transporting activity, internally localized short PINs are more likely to regulate intracellular auxin homeostasis. This study demonstrates that the intracellularly localized PIN8 in the root vasculature is a positive factor in LR formation. Furthermore, ectopic expression of intercellular auxin carriers and auxin signaling components in the *PIN8* domain supports the idea that PIN8-mediated intracellular auxin accumulation, as well as the following enhanced auxin signaling, are involved in LR development.

## Materials and Methods

### Plant Materials and Growth Conditions

Plants of *A. thaliana* ecotype Columbia (Col-0) were used as the wild type (WT) and to generate transgenics, unless otherwise stated. *Arabidopsis* plants were transformed with *Agrobacterium tumefaciens* strain C58C1 carrying a specific construct (described below) using the floral dip method. The transformed plants were selected on media containing hygromycin (30 µg ml^−1^). All seeds were grown in plates containing 4.3 g L^−1^ Murashige and Skoog (MS) nutrient mix (Duchefa, the Netherlands), 1% sucrose, 0.5 g L^−1^ MES (pH 5.7), and 0.8% agarose. All seeds were cold stratified at 4°C for 3 days and germinated at 23°C under 16 h light/8 h dark photoperiod and fluorescent light bulbs (FHF 32SS-EXD; Kumho Electric, Korea) with a light intensity of 130 µmol m^–2^ s^–1^. The *pin8* mutant seeds were purchased from the *Arabidopsis* stock center (http://www.Arabidopsis.org/). The *pin8* null mutation was confirmed by reverse transcription (RT) PCR using *PIN8*-specific primers (**Supplementary Table S1**), with *ACTIN2* as the loading control (**Supplementary Figure S1**).

### Observation of Lateral Roots

LRs and LR primordia were observed with the 9-day-old seedlings after germination unless stated otherwise. The number of LR is the number of emerged LR. LR density was estimated by dividing the emerged LR number by the primary root length in centimeter. LR primordia were observed after clearing process of the root with modification of the previous method (Dubrovsky et al., 2009). Seedlings were treated sequentially with 4% formaldehyde in 0.025 M phosphate buffer (pH 7.2) at 4°C for 16 h, with 30% (v/v) glycerol containing 2% (v/v) DMSO at room temperature for 1.5 h, and with 4 M NaI and 8 mM Na_2_S_2_O_3_ in 65% (v/v) glycerol containing 2% (v/v) DMSO at room temperature for 1.5 h. LR primordia were digitally photographed under a stereomicroscope (Leica MZ FLIII) at 63× magnification.

### Plasmid Construction

The 1.8 kb *PIN8* promoter (*ProPIN8*) was amplified from *Arabidopsis* genomic DNA using sequence-specific primers (**Supplemental Table S1**) and cloned into the binary vector *pCAMBIA1300-NOS* with modified cloning sites (Lee et al., 2010), as described previously (Ganguly et al., 2012). The *PIN8:green fluorescent protein* (*GFP*) fusion was excised from the respective *ProE7* version (Ganguly et al., 2010) and inserted downstream of *ProPIIN8* using *Xho*I and *Xba*I restriction enzymes. To generate *ProPIN8:β-glucuronidase* (*GUS*) fusion, *ProPIIN8* was cloned into the *pBI101* vector using *Hin*dIII and *Sal*1 restriction enzymes. To generate the *ProPIN8:AUX1:yellow fluorescence protein* (*YFP*) construct, the *AUX1:YFP* fragment was excised from the *ProE7:AUX1:YFP* construct (Cho et al., 2007) using *Xho*I and *Xba*I enzymes and inserted downstream of *ProPIN8*. To generate *ProPIN8:PIN2:GFP* and *ProPIN8:PIN3:GFP* constructs, *PIN : GFP* complementary DNA (cDNA) was obtained from *ProE7:PIN2:GFP* and *ProE7:PIN3:GFP* transgenic lines (Lee and Cho, 2006; Ganguly et al., 2010) and inserted downstream of *ProPIN8* using *Sal*I and *Mlu*I restriction enzymes. To generate the *ProPIN8:PIN5:GFP* construct, the cDNA of *PIN5:GFP* was obtained from the *ProE7:PIN5:GFP* transgenic line (Ganguly et al., 2010) and inserted downstream of *ProPIN8* using *Apa*I and *Sac*I restriction enzymes.

An 870 bp fragment of the *GATA23* promoter (*ProGATA23*; -870 to -1 bp relative to the start codon) was amplified from *Arabidopsis* genomic DNA by PCR and cloned into the binary vector *pCAMBIA1300-NOS* using *Hin*dIII and *Sal*I restriction enzymes. To generate *ProGATA23:PIN1:GFP* and *ProGATA23:PIN3:GFP* constructs, the *PIN : GFP* cDNA was amplified from *ProE7:PIN1:GFP* and *ProE7:PIN3:GFP* transgenic lines (Ganguly et al., 2010; Sasayama et al., 2013) and inserted downstream of *ProGATA23* using *Sal*I and *Mlu*I restriction enzymes. To generate *ProGATA23:PIN5:GFP* and *ProGATA23:PIN8:GFP* constructs, *PIN5:GFP* and *PIN8:GFP* fragments were released from their respective *ProE7* versions (Ganguly et al., 2010) and inserted downstream of *ProGATA23* using *Xho*I and *Xba*I restriction enzymes. To generate the *ProGATA23:AXR2-1* construct, the *AXR2-1* fragment was released from the *ProE7* version (Won et al., 2009) and inserted downstream of *ProGATA23* using *Xma*I and *Xba*I restriction enzymes.

### β-Glucuronidase Histochemical Analysis

Nine-day-old seedlings were incubated in GUS reaction buffer (1 mM 5-bromo-4-chloro-3-indoyl-β-d-glucuronic acid cyclohexylammonium salt, 0.1 M NaH_2_PO_4_, 0.01 M EDTA, 0.1% Triton-X, and 0.5 mM potassium ferri- and ferrocyanide [pH 7]) at 37°C for 48 h. The stained seedlings were cleared in 70% ethanol for 1 h and then photographed under a stereomicroscope (Leica MZ FLIII).

### Microscopic Observation and Quantification of PIN8:GFP Fluorescence Signal

LRs were photographed digitally under a stereomicroscope (Leica MZ FLIII) at 60× magnification. GFP (green) and FM4-64 (red) fluorescence were observed using an LSM700 confocal laser scanning microscope (Carl Zeiss) using 488/490–555 and 555/640 nm excitation/emission filter sets, respectively. To determine the localization of the PIN : GFP fusion protein, 9-day-old seedlings were treated with FM4-64 and then incubated in half-strength liquid MS medium. The PIN8:GFP signal was quantified using the histogram function of Adobe Photoshop (Adobe Systems), as described previously (Won et al., 2009). For PIN8:GFP observation in the plasmolyzed epidermal cells of the cotyledon, 3-day-old WT or *ProPIN8:PIN8:GFP* transformant (in *pin8*) seedlings were treated with 0.05% Tween 20 for 2 h and then with 1 M mannitol for 5 to 10 min.

### Ribonucleic Acid Isolation and Quantitative Reverse Transcription Polymerase Chain Reaction

Total RNA was isolated from the roots of 9-day-old seedlings using the RNeasy Plant Mini Kit (Qiagen) and used for cDNA synthesis as described previously (Lee and Cho, 2006). Then, quantitative real-time (qRT)-PCR was performed on a CFX Connect Real-Time PCR Detection System (Bio-Rad) using amfiSure qGreen Q-PCR Master mix without ROX (Applied GenDEOT). Gene expression levels were normalized relative to *ACTIN2* expression. The qRT-PCRs were performed using three independent RNA preparations, each with three technical replicates. Primers used for qRT-PCR are listed in **Supplementary Table S1**.

### Accession Numbers

Sequence data and mutant information from this article can be found in the Arabidopsis Genome Initiative databases under the following accession numbers: AT1G70940 (*PIN3*), AT1G73590 (*PIN1*), AT1G77690 (*LAX3*), AT2G36010 (*E2Fa*), AT2G38120 (*AUX1*), AT2G42430 (*LBD16*), AT2G45420 (*LBD18*), AT3G187800 (*ACTIN2*), AT3G23050 (*AXR2*), AT3G58190 (*LBD29*), AT4G01630 (*EXPA17*), AT5G06080 (*LBD33*), AT5G15100 (*PIN8*), AT5G16530 (*PIN5*), AT5G26930 (*GATA23*), AT5G56320 (*EXPA14*), AT5G57090 (*PIN2*), and SALK_107965 (*pin8*).

## Results

### PIN8 Affects Lateral Root Development

**Figure 1:**
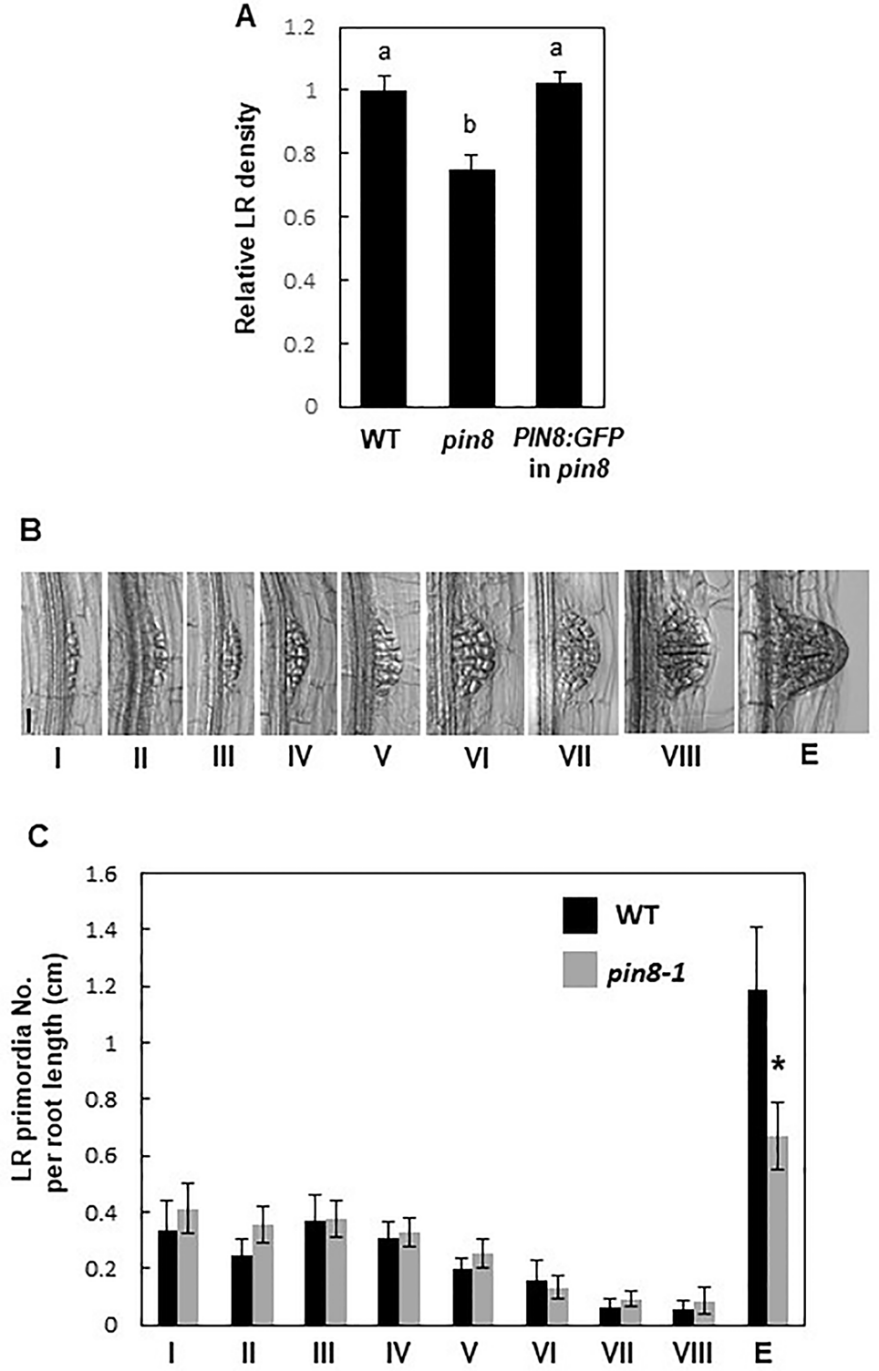
PIN8 affects lateral root (LR) development. **(A)** LR densities (number of emerged LRs per cm of the root) of the wild type (WT), *pin8* mutant, and *pin8*-complementation line expressing *ProPIN8:PIN8:GFP*. Data represent mean ± standard error [SE; *n* = 142–261 seedlings; from seven independent lines for *ProPIN8:PIN8:GFP* in *pin8* (**Supplementary Figure S2C**)]. Statistically significant differences were determined using one-way analysis of variance (ANOVA) with Tukey's unequal N honest significant difference (HSD) *post hoc* test and are denoted with different letters (*P* < 0.05). **(B)** Representative images of LR primordia at different developmental stages. Scale bar = 40 µm. **(C)** Distribution of LR primordia at different developmental stages. Data represent mean ± SE (*n* = 17–20 seedlings). “E” in (**B, C**) indicates the emerged LR primordia. Significant differences compared with WT are indicated using asterisks (*P < 0.05; Student's *t*-test).

The loss-of-function *pin8* mutant (**Supplementary Figure S1**) showed a significant decrease (~25%) in LR density compared with the WT (**Figure 1A**; **Supplementary Figures S2** and **S3**). However, the *PIN8* defect did not significantly affect the primary root growth (**Supplementary Figure S2A**). Complementation of the *pin8* mutant with *PIN8* fused to the *GFP* gene under the control of its native promoter (*ProPIN8:PIN8:GFP*) restored the LR density of the *pin8* mutant to the WT level, indicating that PIN8 affects LR development (**Figure 1A**). Analysis of LR development (from stage I–VIII) and LR emergence revealed that the LR defect in the *pin8* mutant was at the emergence stage (**Figures 1B, C**), implying that PIN8 mainly regulates LR emergence from the root.

**Figure 2:**
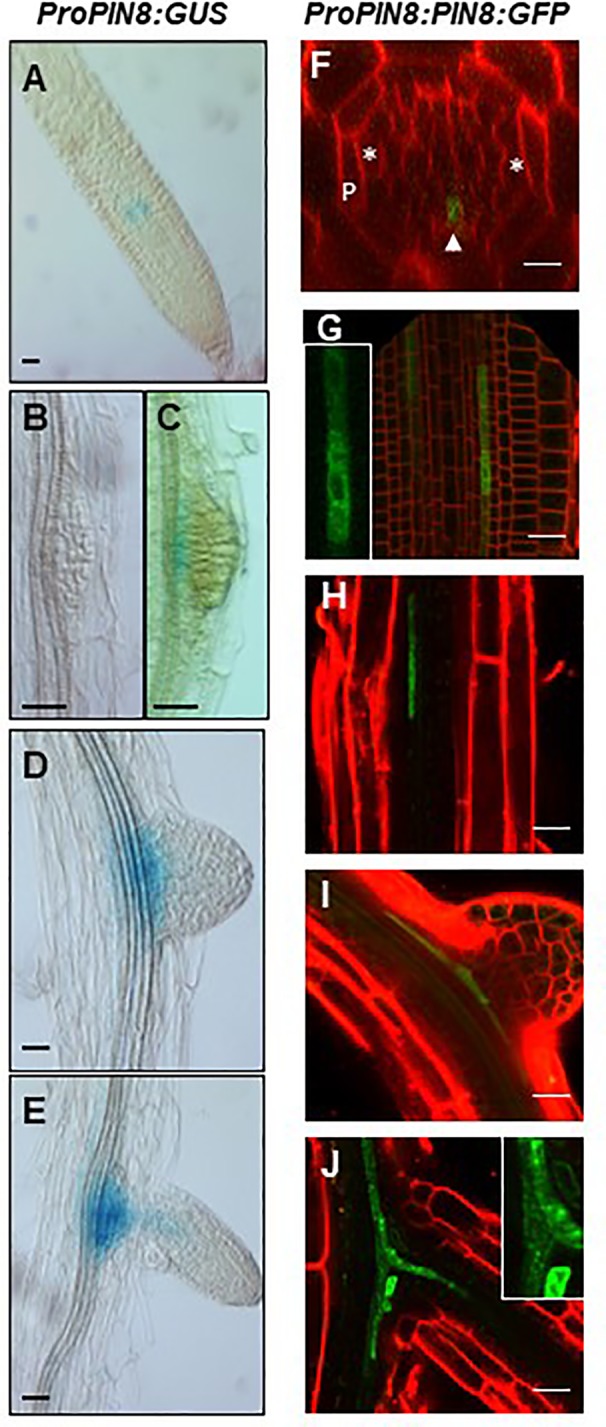
Expression pattern of *PIN8* in the root. **(A–E)** Expression of *ProPIN8:GUS* in the root meristem region **(A)** and around the developing lateral root (LR) primordia **(B–E)**. **(F–J)** Expression of *ProPIN8:PIN8:GFP* in the root meristem **(F–G)** and elongation/maturation **(H)** regions and around the developing LR primordia **(I–J)**. Asterisks, “P,” and the arrowhead in **F** (a confocal z-section image of the root basal meristem region) indicate xylem-pole positions, pericycle, *and PIN8:GFP* signal, respectively. The insets of (**G, J**) are the enlarged images of *PIN8:GFP*-expressing cells. Red signals represent FM4-64 staining. Scale bars = 20 µm.

To determine the spatial expression profile of *PIN8* during LR development at both transcript and protein levels, the expression of *GUS* and *GFP* reporter genes was monitored in *ProPIN8:GUS* and *ProPIN8:PIN8:GFP* transgenic lines (**Figure 2**). *PIN8* was expressed in the vasculature cells of the basal meristem and elongation/maturation regions in a punctate pattern along the vasculature, which is reminiscent of the dispersed LR primordia and their ancestral cells along the root axis (**Figures 2A, F–H**). These *PIN8*-expressing vascular cells were present in the phloem rather than the xylem because the *PIN8*-expressing cells axis was perpendicular to the xylem-pole axis (**Figure 2F**). Robust *PIN8* expression was observed in the vasculature at the base of the LR primordium (**Figures 2C–E, I–J**). However, the expression of *PIN8* was not detected in this region before stage VIII of LR development (**Figures 2B, C**), suggesting that PIN8 is involved in LR primordium development mainly at a late stage. When the emerged LR started elongating, *PIN8* expression in the primary root started extending to the LR vasculature (**Figures 2E, J**).

We previously showed that the PIN8 protein, when expressed ectopically in root hair cells using the root hair-specific *ProE7*, tobacco BY-2 cells using the dexamethasone-inducible promoter, and root epidermal cells using *ProPIN2*, localizes to both the cytoplasm and PM (Ganguly et al., 2010; Ganguly et al., 2014). However, in this study, the PIN8 protein predominantly localized to the intracellular compartments in the root vascular cells when expressed in its native domain (**Figures 2F–J**). The predominant intracellular localization of PIN8 in the vasculature indicates that PIN8 plays a distinct role in cellular auxin dynamics, unlike long PINs that predominantly localize to the PM and export auxin out of the cell.

**Figure 3:**
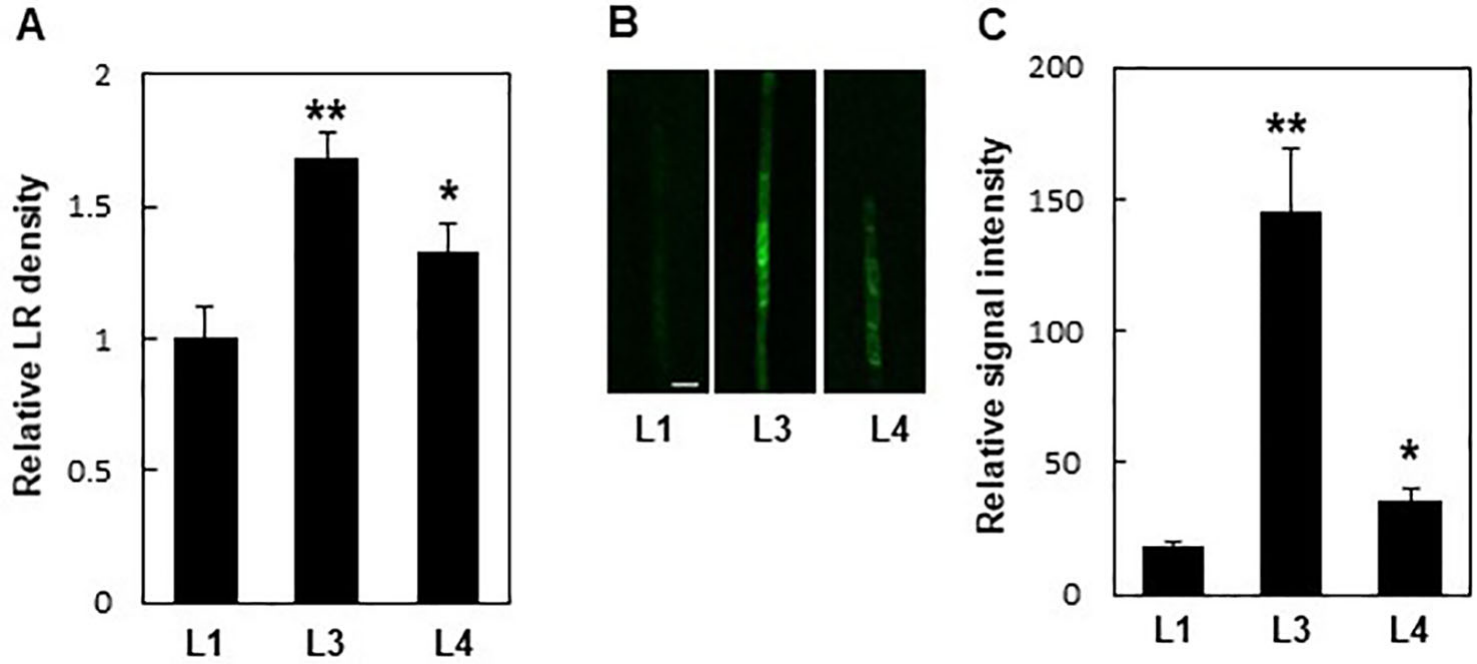
Expression levels of *PIN8* are related to lateral root (LR) density. **(A)** LR densities of three independent *ProPIN8:PIN8:GFP* transgenic lines. Data represent mean ± SE (*n* = 27–66 seedlings). **(B)** Confocal images of the *PIN8:GFP* signal in the root vasculature of *ProPIN8::PIN8:GFP* transgenic lines. Scale bar = 20 µm. **(C)** Relative *PIN8:GFP* signal in *ProPIN8:PIN8:GFP* lines. Data represent mean ± SE (*n* = 5 seedlings). In (**A, C**), significant differences compared with L1 intensity are indicated using asterisks (*P < 0.05, **P < 0.005; Student's *t*-test).

Additionally, PIN8 mediated LR development in a dose-dependent manner. Monitoring LR density in independent *ProPIN8:PIN8:GFP* transgenic lines showing different *PIN8:GFP* expression levels revealed that lines with higher *PIN8* expression produced more LRs (**Figure 3**; **Supplementary Figure S4**). This result further confirms that PIN8 plays a positive role in LR development.

### PIN8-Mediated Lateral Root Development Requires Auxin Influx in PIN8-Expressing Cells

PM-localized PINs exhibit auxin export activity, which promotes intercellular auxin transport but lowers auxin level in the cell where they are acting. Conversely, the internally localized PINs are likely to increase intracellular auxin levels for nuclear auxin signaling and response as shown by the enhanced root hair growth in the *ProE7:PIN5:GFP* transformant (Ganguly et al., 2010). Because PIN8 proteins are mostly distributed among the intracellular compartments in the root vasculature, we were curious to know whether PIN8 promotes LR development by increasing internal auxin levels. Therefore, we expressed genes encoding two long PINs (PIN2 and PIN3), a short PIN (PIN5), and an auxin influx carrier (AUX1) in the *PIN8* domain under the control of the *PIN8* promoter in WT or in the *pin8* mutant background. We hypothesized that if PIN8-mediated promotion of LR development results from PIN8-mediated increase of cellular (thus nuclear) auxin level, then auxin-exporting long PINs and auxin-importing AUX1 in the *PIN8* domain would show opposite effects on LR development, i.e., inhibition and promotion of LR development, respectively. In this context, the LR-defective *pin8* mutant phenotype would be rescued by the expression of *AUX1* but not by that of *PIN2* or *PIN3*.

**Figure 4:**
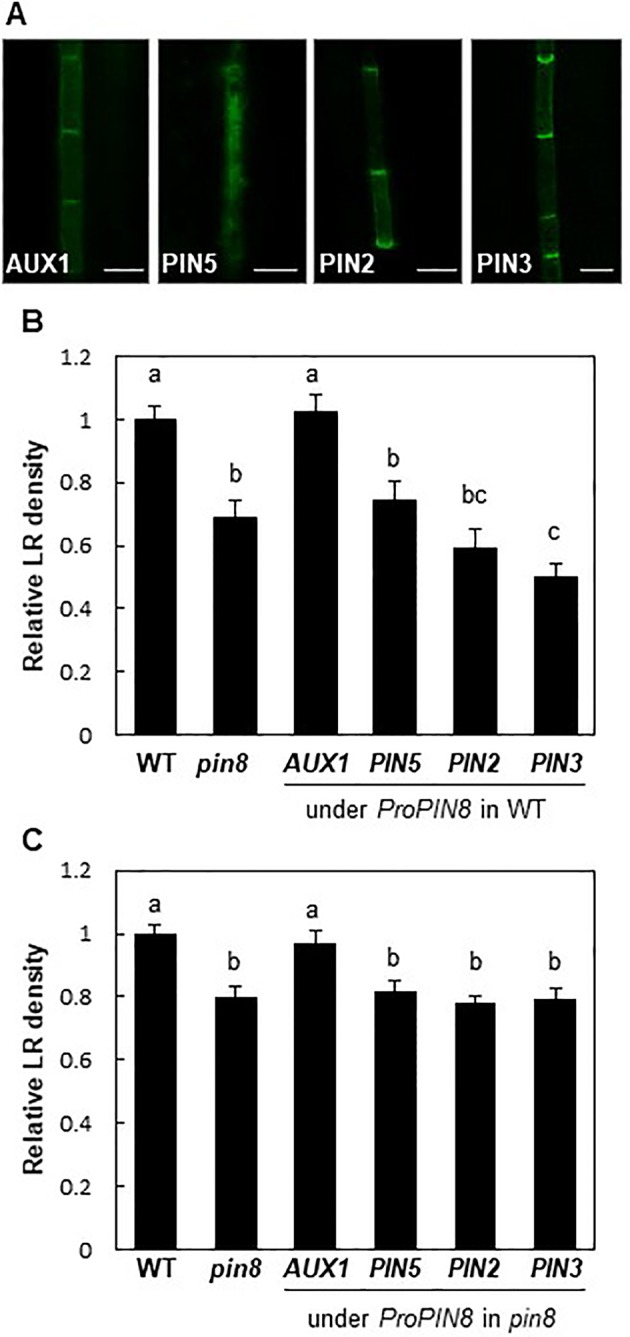
Effect of auxin transporters on lateral root (LR) development when expressed in the *PIN8* domain. **(A)** Confocal images of the root vascular cells expressing *AUX1:YFP*, *PIN5:GFP*, *PIN2:GFP*, and *PIN3:GFP* under the control of the *PIN8* promoter (*ProPIN8*). Scale bar = 10 µm. **(B, C)** LR densities of the WT, *pin8* mutant, and transgenic lines expressing *ProPIN8*-driven auxin transporter genes in the WT **(B)** and *pin8* mutant **(C)** backgrounds. Data represent mean ± SE [*n* = 29–37 seedlings for **B** and 61–68 seedlings for **C**; in case of transgenics, 5 independent lines per construct were observed (**Supplementary Figures S5E, F**)]. Statistically significant differences are denoted with different letters [*P* < 0.05; one-way ANOVA with Tukey's unequal N honest significant difference (HSD) *post hoc* test].

AUX1 and long PINs exhibited the typical PM localization with an apical/basal polarity in the PIN8 domain (**Figure 4A**). In the WT background, long PINs significantly inhibited LR development, similar to that in the *pin8* mutant, whereas AUX1 did not show a noticeable effect (**Figure 4B**; **Supplementary Figure S5**). Conversely, in the *pin8* mutant background, AUX1, but not PIN2 or PIN3, was able to complement the LR defect (**Figure 4C**; **Supplementary Figure S5**). Considering that AUX1 and long PINs in the PM are responsible for cellular auxin import and export and following increase and decrease of nuclear auxin levels, respectively, these data suggest that internally localized PIN8 is likely to increase the nuclear auxin level *via* intracellular auxin translocation, which could be positively operating for LR development. In addition to its complementary function during LR emergence, AUX1 also somewhat enhanced primordium formation of stage I and II (**Supplementary Figure S6**), raising a possibility that ectopically expressed AUX1 in the *PIN8* domain has an additional function in the early LR development where PIN8 does not have it. Although it is speculative, PIN8-mediated intracellular auxin translocation during early LR development could be insufficient to enhance nuclear auxin signaling whereas AUX1-mediated cellular auxin import is sufficient for it.

PIN5, another short PIN, was shown to localize to intracellular compartments and regulate intracellular auxin translocation (Mravec et al., 2009; Ganguly et al., 2010; **Figure 4A**). When expressed in the PIN8 domain of the *pin8* mutant, PIN5 failed to restore the mutant phenotype, unlike AUX1 (**Figure 4C**). Moreover, *PIN5* expression in the PIN8 domain of WT plants also decreased the LR density (**Figure 4B**; **Supplementary Figure S5**). This suggests that although both PIN8 and PIN5 are localized to intracellular compartments, their auxin-transporting functions could be different at the subcellular level, similar to their opposite functions during *Arabidopsis* leaf vein development (Sawchuk et al., 2013; Verna et al., 2015).

### PIN8 Negatively Affects Lateral Root Development in the GATA23 Domain

**Figure 5:**
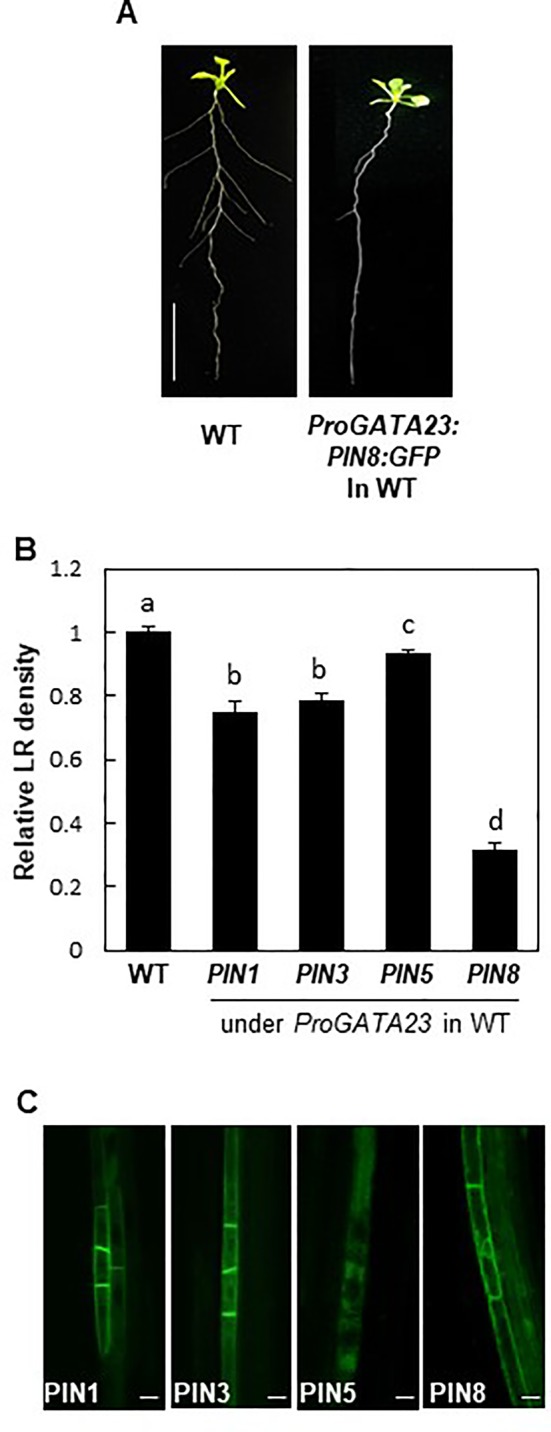
Effect of PINs on lateral root (LR) development when expressed in the *GATA23* domain. **(A)** Root phenotypes of the wild type (WT) and *ProGATA23:PIN8:GFP* transgenic line. **(B)** LR densities of the WT and *ProGATA23:PINs : GFP* transgenic lines. Data represent mean ± SE [*n* = 89–694 seedlings; in case of transgenics, 3–12 independent lines per construct were observed (**Supplementary Figure S7G**)]. Statistically significant differences are denoted with different letters [*P* < 0.05; one-way ANOVA with Tukey's unequal N honest significant difference (HSD) *post hoc* test]. **(C)** Confocal images of pericycle cells expressing *PIN : GFP* fusions under the control of *ProGATA23*. Scale bar = 20 µm.

To obtain further insights into the function of PIN8 in LR development, we ectopically expressed *PIN8* in pericycle cells under the control of *ProGATA23*. The GATA23 transcription factor, which is specifically expressed in LR founder and primordial cells of the pericycle, is one of the key modulators of LR development (De Rybel et al., 2010). *GATA23*-expressing pericycle cells adjacent to the xylem pole accumulate auxin and then drive auxin signaling for LR development. We anticipated that the *GATA23*-expressing cell could serve as a model site for the characterization of cellular auxin-transporting property of PIN8 for LR development. It is conceivable that if PIN8 plays as an intracellular auxin translocator to the nucleus in the *GATA23* domain similarly in the PIN8 domain, its ectopic expression in the *GATA23* domain would enhance LR development. Intriguingly, *PIN8* expression in the pericycle cells greatly decreased the LR density (~70%) (**Figures 5A, B**; **Supplementary Figure S7**). The expression of *PIN1* and *PIN3* under the control of *ProGATA23* also decreased the LR density significantly, whereas the expression of *PIN5* decreased the LR density only marginally (**Figure 5B**; **Supplementary Figure S7**). In the *GATA23*-expressing pericycle cells, both long PINs (PIN1 and PIN3) showed a distinct apical/basal polar localization in the PM, whereas PIN8 showed a dual internal and PM localization pattern (**Figure 5C**). This PIN8 localization pattern was consistent in independent transgenic lines expressing different PIN8 levels (**Supplementary Figure S8**). By contrast, PIN5 predominantly localized to the internal compartments. This result suggests that PIN8 most likely acts as a cellular auxin exporter in the PM of the *GATA23*-expressing pericycle cells and thus shows the inhibitory effect on LR development. Because GATA23 plays in multiple stages from founder cell specification to LR initiation, this *ProGATA23:PIN8*-mediated LR inhibition could be a result from multiple action of PIN8 in the *GATA23* domain.

### Auxin Signaling in the PIN8 Domain Is Required for Lateral Root Development

**Figure 6:**
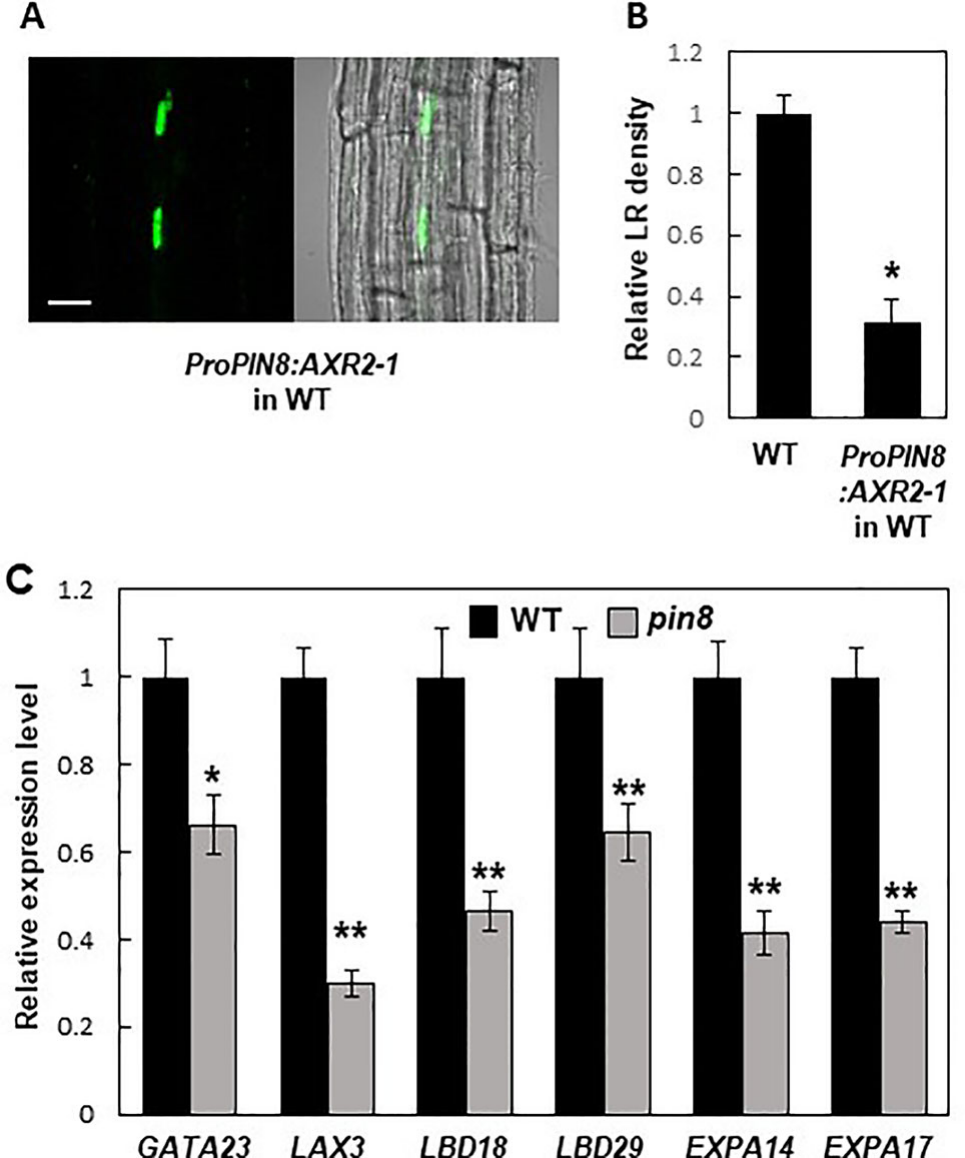
Auxin signaling in the *PIN8* domain is required for lateral root (LR) development. **(A)** Confocal images [fluorescence (left) and bright field (right)] showing the *AXR2-1:GFP* signal in the *PIN8* domain of the root. Scale bar = 20 µm. **(B)** LR densities of the wild type (WT) and *ProPIN8:AXR2-1:GFP* transgenic line. Data represent mean ± SE [*n* = 35–36; from 3 independent lines for *ProPIN8:AXR2-1:GFP* (**Supplementary Figure S9C**)]. Significant differences compared with the WT are indicated using an asterisk (*P < 10^−9^; Student's *t*-test). **(C)** Relative transcript levels of LR-related genes in the WT and *pin8* mutant. Data represent mean ± SE of three biological replications. Significant differences compared with the WT are indicated using asterisks (*P < 0.05, **P < 0.01; Student's *t*-test).

*PIN8* is expressed in the phloem, whereas LR development occurs at the pericycle near the xylem. This spatial discrepancy between the *PIN8* expression zone and the LR development zone led us to a question how PIN8 remotely affects LR development. Because PIN8 is likely to facilitate cellular accumulation of auxin in its native domain, it is conceivable that the nuclear auxin signaling could be activated in the *PIN8* domain, which then remotely affects LR development in the xylem-pole pericycle. To test this possibility, we introduced a dominant negative version (AXR2-1) of AXR2, an Aux/IAA transcriptional repressor of auxin-responsive genes, into the *PIN8*-expressing phloem cells under *ProPIN8* (**Figure 6A**). Previously, we showed that root hair-specific expression of *AXR2-1* completely inhibited root hair growth, which requires positive auxin signaling (Won et al., 2009; Mangano et al., 2017), suggesting that AXR2-1 is a strong suppressor of auxin signaling. Expression of *AXR2-1* in the *PIN8*-expressing phloem cells greatly reduced LR density (**Figure 6B**; **Supplementary Figure S9**). This result suggests that auxin signaling in the PIN8-acting phloem cells is important for LR development. However, since the AXR2-1 expression in the *PIN8* domain also inhibited primary root growth (**Supplementary Figure S9A**), we do not exclude the possibility that AXR2-1 affects LR development *via* the process requiring positive auxin signaling other than the LR emerging process. Intriguingly, the AXR2-1 expression rather increased the density of stage I and II primordia (**Supplementary Figure S9D**). Considering its negative function in auxin signaling, AXR2-1 might block the progress after stage II, which would result in accumulation of stage I and II primordia.

To investigate whether PIN8 also affects LR-related nuclear auxin signaling, we examined the transcript levels of auxin- and LR-related genes in the WT and *pin8* mutant. Our data showed that *GATA23* expression was significantly reduced in the *pin8* mutant (**Figure 6C**). Transcript levels of both *LAX3* (encoding an auxin influx carrier) and *LateralOrganBoundariesDomain18* (*LBD18*, downstream target of LAX3) were also considerably decreased in the *pin8* mutant compared with the WT (**Figure 6C**). LBD transcription factors are key regulators of genes involved in LR development (Okushima et al., 2005; Okushima et al., 2007; Berckmans et al., 2011; Goh et al., 2012; Lee et al., 2015). *LBD29*, which encodes a transcriptional activator of *LAX3* (Okushima et al., 2007; Porco et al., 2016), also showed reduced transcript level in the *pin8* mutant compared with the WT (**Figure 6C**). The LBD18-upregulated genes, *EXPANSIN A14* (*EXPA14*) and *EXPA17* which encode cell-wall loosening proteins (Lee et al., 2013; Lee and Kim, 2013; Lee et al., 2015), also showed reduced expression in the *pin8* mutant compared with the WT (**Figure 6C**). LBD18, along with LBD33, activates the transcription of *EF2a* for LR initiation at the sites where the EF2a transcription factor is required for asymmetric cell division of the LR founder cell (Berckmans et al., 2011; Goh et al., 2012). LBD16 is also involved in this LR initiation stage (Goh et al., 2012). In our gene expression analysis, most of the genes engaged in early LR development did not show a significant difference in expression levels between the WT and *pin8* mutant (**Supplementary Figure S10**), although the expression of *GATA23* was suppressed in the mutant background (**Figure 6C**). Together, these results suggest that the PIN8-mediated change in auxin homeostasis in phloem cells is involved in the transcriptional regulation of a battery of genes involved in LR development at the emergence stage.

## Discussion

Auxin is the key hormonal factor involved in almost all major steps of LR development. A subtle coordination between auxin transport and auxin signaling orchestrates the determination of LR primordial cell fates and regulates LR formation and expansion. Although previous studies have identified a plethora of factors involved in LR development (Du and Scheres, 2018), the role of auxin transporters in intra- and intercellular auxin movement during LR development has not been fully elucidated. Previous studies have shown than PIN8 functions as an auxin carrier required for pollen development and pollen tube growth (Dal Bosco et al., 2012; Ding et al., 2012). In pollens, the ER-localized PIN8 regulates intracellular auxin homeostasis to promote male gametophyte development in *Arabidopsis* (Ding et al., 2012). In this study, we showed that PIN8 is involved in LR development, possibly by modulating intracellular auxin translocation at the LR emergence zone.

The root pericycle consists of two cell types adjacent to the xylem and phloem poles, with different cytological features and cell fates (Beeckman et al., 2001; Himanen et al., 2004; Parizot et al., 2008); phloem-pole-pericycle cells (PPP) are quiescent, whereas xylem-pole-pericycle cells are semi-meristematic and undergo cell division to give rise to the LRFCs. Relatively little is known about how the phloem cells function during LR development. Phloem cells have been implicated in the regulation of LR positioning, although the mechanism remains unknown (Notaguchi et al., 2012). Interestingly, *IAA18* messenger RNA (mRNA), which is synthesized in the vasculature of leaves and mature root, is translocated through the phloem to the basal meristem (Notaguchi et al., 2012). IAA18 is an important auxin signaling component, which inhibits the function of ARF7 and ARF19. Auxin-induced degradation of IAA18 is critical for the de-repression of *ARF7* and *ARF19* genes, and the free forms of ARF7 and ARF19 initiate downstream transcriptional activation of the factors required for LR founder cell specification (Uehara et al., 2008). The spatial coincidence between *PIN8* expression and *IAA18* mRNA translocation in the phloem prompts us to speculate that PIN8-mediated alteration of auxin homeostasis could affect *IAA18* mRNA stability.

The late expression of *PIN8* from LR developmental stage VIII onward indicates that PIN8 plays a more active role in LR emergence. This is supported by our data showing no significant changes in transcript levels of early LR primordium-forming genes, such as *EF2a*, *LBD16*, and *LBD33*, in the *pin8* mutant (**Supplementary Figure S10**). However, genes required mainly for LR emergence, such as *LAX3*, *EXPA14*, *EXPA17*, and *LBD18*, showed decreased expression in *pin8* (**Figure 6C**), consistent with the major LR emergence defect of the mutant (**Figure 1C**). Together, these data suggest that the developmentally regulated *PIN8* expression is implicated in local cellular auxin homeostasis required for LR emergence. On the other hand, the role of early expressing PIN8 in the developing phloem of the basal meristem is not obvious. The *ProPIN8:PIN2* and *PIN3* (in *pin8*) transformants, which are as defective in LR development as in the *pin8* mutant, did not show noticeable morphological defects in phloem cells (**Figure 4A**).

One of the most intriguing findings of this study is that the auxin influx carrier AUX1 was able to complement the *pin8* mutant when expressed in the *PIN8* domain. AUX1 facilitates auxin influx from the apoplast to the cytosol, which increases intracellular auxin concentration and ultimately stimulates nuclear auxin signaling in the AUX1-acting cell (Bennett et al., 1996; Yang et al., 2006). On the other hand, the predominantly PM-localized long PINs (PIN1–4 and PIN7) facilitate auxin efflux from the cytosol to the apoplast (Ganguly et al., 2010). Interestingly, none of the PM-localized efflux carriers, such as PIN2 and PIN3, or the internally localized PIN5 protein were able to complement the LR formation defect of the *pin8* mutant. These observations suggest that PIN8 is functionally unique among long and short PINs. It is striking that PIN8 showed an opposite phenotypic effect on LR development to that of PIN5, although both of these short PINs localize to internal compartments (**Figure 4**). This opposite function of PIN8 and PIN5 in LR development is reminiscent of the antagonistic role of these two PINs in leaf vein development in which PIN8 promotes intracellular auxin activity whereas PIN5 does the opposite function (Sawchuk et al., 2013; Verna et al., 2015). However, because the intracellular distribution patterns of PIN5 and PIN8 are different (Ganguly et al., 2010), PIN8-catalyzed intracellular auxin translocation may lead to a different result from that of PIN5. Similar to AUX1, PIN8 may increase the cytosolic auxin pool, but by catalyzing auxin reflux out of the ER, which would require a topologically opposite directionality of auxin transport to that of long PINs. Additionally, PIN8 transports not only indole-3-acetic acid (IAA) but also its precursor, indole-3-butyric acid (IBA) (Ding et al., 2012). Whether PIN8 has a preference for a particular auxin species is an interesting question that remains to be answered. Preferential transport of any particular auxin type may alter the internal auxin homeostasis of the cell, thereby modifying the cellular transcriptional response.

Another interesting aspect of PIN8 is its promiscuous subcellular and functional behaviors in different cell types. In this study, endogenous *PIN8* promoted the development of LRs, which is most likely due to the increased auxin level and signaling in the *PIN8* domain (**Figures 1**, **4**, and **6**). However, ectopically expressed *PIN8* in pericycle cells inhibited LR development (**Figure 5**). Considering that both domains require the accumulation of cellular auxin for LR development, these results imply that PIN8 could facilitate either accumulation or export of auxin in the cell, depending on the cell type. One of the possible explanations for this phenomenon is the different subcellular localization of PIN8 in different cell types. When observed in its native domain, while PIN8 intracellularly localizes in the root vascular cells, it obviously localizes to the PM of cotyledon epidermal cells (**Supplementary Figure S11**). Because the *pin8* mutant cotyledons did not show notable phenotypic changes in, the PIN8 function in this tissue is not clear. Ectopic expression studies also showed that PIN8 can localize to either intracellular compartments or PM depending on the cell type and developmental stage (Ganguly et al., 2010; Dal Bosco et al., 2012; Ding et al., 2012; Ganguly et al., 2014). PM-localized PIN8 is expected to export auxin out of the cell, which lowers nuclear auxin availability for auxin responses. Internally localized PIN8 may either decrease or increase auxin availability in the nucleus, depending on the internal compartment where PIN8 resides. If PIN8 localizes to the ER, it may supply auxin to the nucleus. However, if PIN8 is present in other intracellular compartments, it may sequestrate auxin within those compartments, resulting in low auxin availability in the nucleus. Additionally, if PIN8 is localized in secretory vesicles, it may export auxin out of the cell similarly as seen in the neurotransmitter-secretory pathway (Baluška et al., 2003). In this study, with respect to LR development, we demonstrated that 1) PIN8 predominantly localizes to intracellular compartments; 2) the PIN8-expressing cell requires a positive auxin signaling for LR development; and 3) the auxin influx carrier AUX1 complements PIN8 function. These observations suggest that the internally localized PIN8 is likely to act as a nuclear auxin supplier in cells neighboring the LR primordium.

PIN5, another short PIN, also shows different subcellular localizations in its native domain and when ectopically expressed, depending on the cell type (Ganguly et al., 2014). Taken together, our study suggests that short PINs are involved in diverse auxin responses and intracellular auxin homeostasis by dynamically changing their subcellular localization.

## Data Availability Statement

All datasets generated for this study are included in the article/**Supplementary Material**.

## Author Contributions

AG and H-TC designed the project. HL, AG, RDL, and MP performed the experiments. All authors contributed to the interpretation of the results and the writing of the manuscript and approve the final manuscript.

## Funding

This research was supported by grants from the National Research Foundation (NRF-2017R1E1A1A01075264) and the Next-Generation BioGreen 21 Program (Agricultural Genome Center PJ013300) of the Rural Development Administration. HL was partially supported by the Stadelmann-Lee Scholarship Fund, Seoul National University.

## Conflict of Interest

The authors declare that the research was conducted in the absence of any commercial or financial relationships that could be construed as a potential conflict of interest.
