# Supplementary material for: Intracellularly Localized PIN-FORMED8 Promotes Lateral Root Emergence in Arabidopsis
Source: Front Plant Sci. 2020 Jan 31;10:1808. doi: 10.3389/fpls.2019.01808 (PMC7005106; doi:10.3389/fpls.2019.01808)
Supplement: Supplementary file 1 [file DataSheet_1.pdf]

Table S1. List of primers used in this study

Subject	Primer name[1]	Primer sequence (5' to 3')
PIN8 promoter	proPIN8-Hd-F	CTGCGAAGCTTGTAAAATGTGAAGG
	proPIN8-Sl-R	TATAGTCGACGTTTTTATCAAATTGTACAATAC
PIN8:GFP	PN8-CD-Xh-F	ATTTTCTCGAGATGATCTCCTGGCTCGATATCTAC
	PN8-CD-Xb-R	TAAAATCTAGATCATAGGTCCAATAGAAAATAATATGCCA
PIN1:GFP	PIN1-CD-Sl-F	TATAGTCGACATGATTACGGCGGCGGACTTCTAC
	PIN1-CD-Ml-R	TATAACGCGTTCATAGACCCAAGAGAATGTAGTA
PIN2:GFP	PIN2-CD-Sl-F	TATAGTCGACATGATCACCGGCAAAGACAT
	PIN2-CD-Ml-R	TATAACGCGTTTAAAGCCCCAAAAGAACGTAGTA
PIN3:GFP	PIN3-CD-Sl-F	TATAGTCGACATGATCTCATGGCACGACCTCTAC
	PIN3-CD-Ml-R	TATAACGCGTTTATAACCCGAGTAGAATGTAGTA
PIN5:GFP	PIN5-CD-Ap-F	TATAGGGCCCATGATAAATTGTGGAGATGTTTAC
	PIN5-CD-Sc-R	TATAGAGCTCTCAATGAATAAACTCCAGAG
GATA23 promoter	pGATA23-Hd-F	TATAAAGCTTATAACTTTTCAATAATGG
	pGATA23-Sl-R	TATAGTCGACCAAATAAAAAAAAACAATCTTAGTTC
RT-PCR	PIN8-RT-F	GGATTATCTATATCGGTGTTGC
	PIN8-RT-R	GAAGATGGTAACGCCCTTGC
	ACT2-RT-F	GATCGGTGGTTCCATTCTTG
	ACT2-RT-R	ATCTTGAGAGCTTAGAAACATT
	LAX3-RT-F	TCACCATTGCTTCACTCCTTC
	LAX3-RT-R	AAGCACCATTGTGGTTGGAC
	PG-RT-F	CATCGATGGACGAGGATCA
	PG-RT-R	CCTCAAAGCTGTTGGTTTGG
	LBD33-RT-F	TCGCTGCTATCACCATCTCC
	LBD33-RT-R	GCCATAGTCATCTGATTCACAAACTCC
	GATA23-RT-F	AGTGAGAATGAAAGAAGAGAAGGG
	GATA23-RT-R	GTGGCTGCGAATAATATGAATACC
	EXPA14-RT-F	CAATACCGGAGAGTGGCTTGCC
	EXPA14-RT-R	TTGTTAGATATAACTGTACGGCC
	EXPA17-RT-F	GACCTTTTATGGCGGAAGTGATGCC
	EXPA17-RT-R	TCTCGGTGGGTTGCACCAACCTCC
	LBD18-RT-F	GTGCATAAAGTGTTCGGAGC
	LBD18-RT-R	CTTCGTTGTTGCGTGGCCCA
	E2Fa-RT-F	CGAAGCCTTAACTGTTGACAACC
	E2Fa-RT-R	GCATTTGAGAGAAGCCAGTAGTCC
	LBD16-RT-F	TACAACGGCGGGGACAGGT
	LBD16-RT-R	GCTGCGAATCTTGCTGCTCC
	LBD29-RT-F	GCTAGGCTTCAAGATCCCATC
	LBD29-RT-R	TGTGCTGCTTGTTGCTTTAGA

[1] Abbreviations of restriction enzyme sites in primers: Av, AvrII; Xm, XmaI; Sl, SalI; Xh, Xho1; Xb, XbaI; Hd, HindIII; Ml, MluI; Ap, ApaI; Sc, SacI.

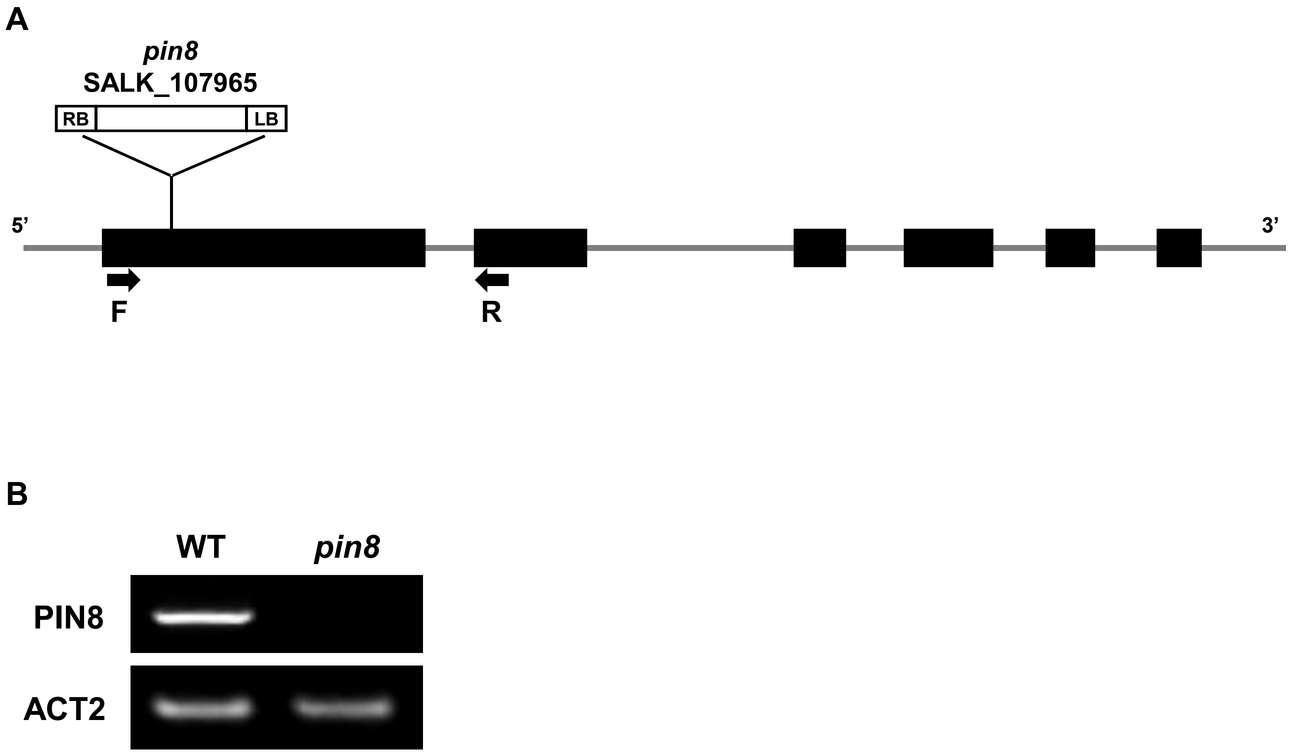

Figure S1. Verification of the *pin8* mutant line. **(A)** A schematic diagram showing T-DNA insertion site in the *pin8* mutant line (SALK_107965). Exons are indicated by black boxes. 'F' and 'R' designate the primer positions for RT-PCR in **B**. **(B)** RT-PCR analysis of RNA from wild type (WT) and *pin8* roots.

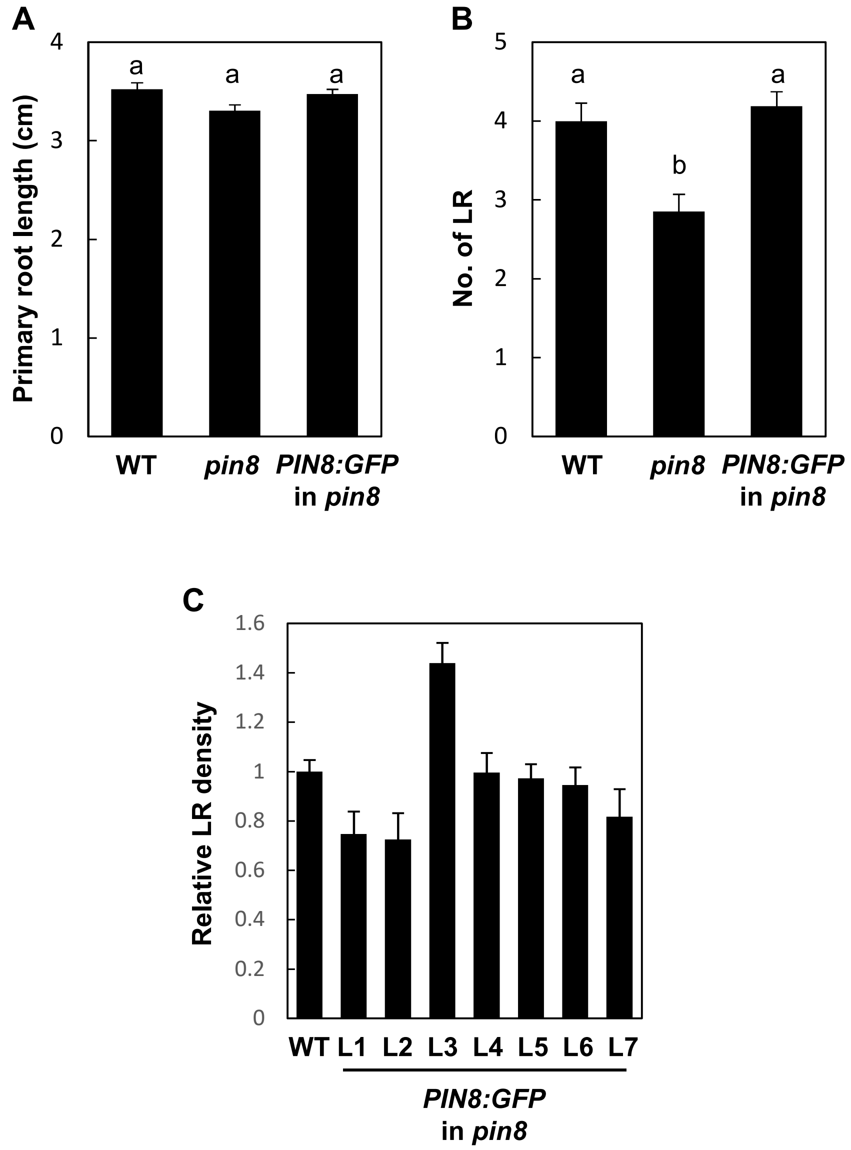

Figure S2. Primary root length **(A)** and number of LR **(B)** of the wild type (WT), *pin8* mutant, and *pin8*-complementation lines expressing *ProPIN8:PIN8:GFP,* and relative LR density of the independent transgenic lines for *ProPIN8:PIN8:GFP* in *pin8* (**C**). Data represent mean ± SE (n = 142–261 seedlings). Statistically significant differences were determined using one-way analysis of variance (ANOVA) with Tukey's unequal N HSD post hoc test and are denoted with different letters ($P < 0.05$).

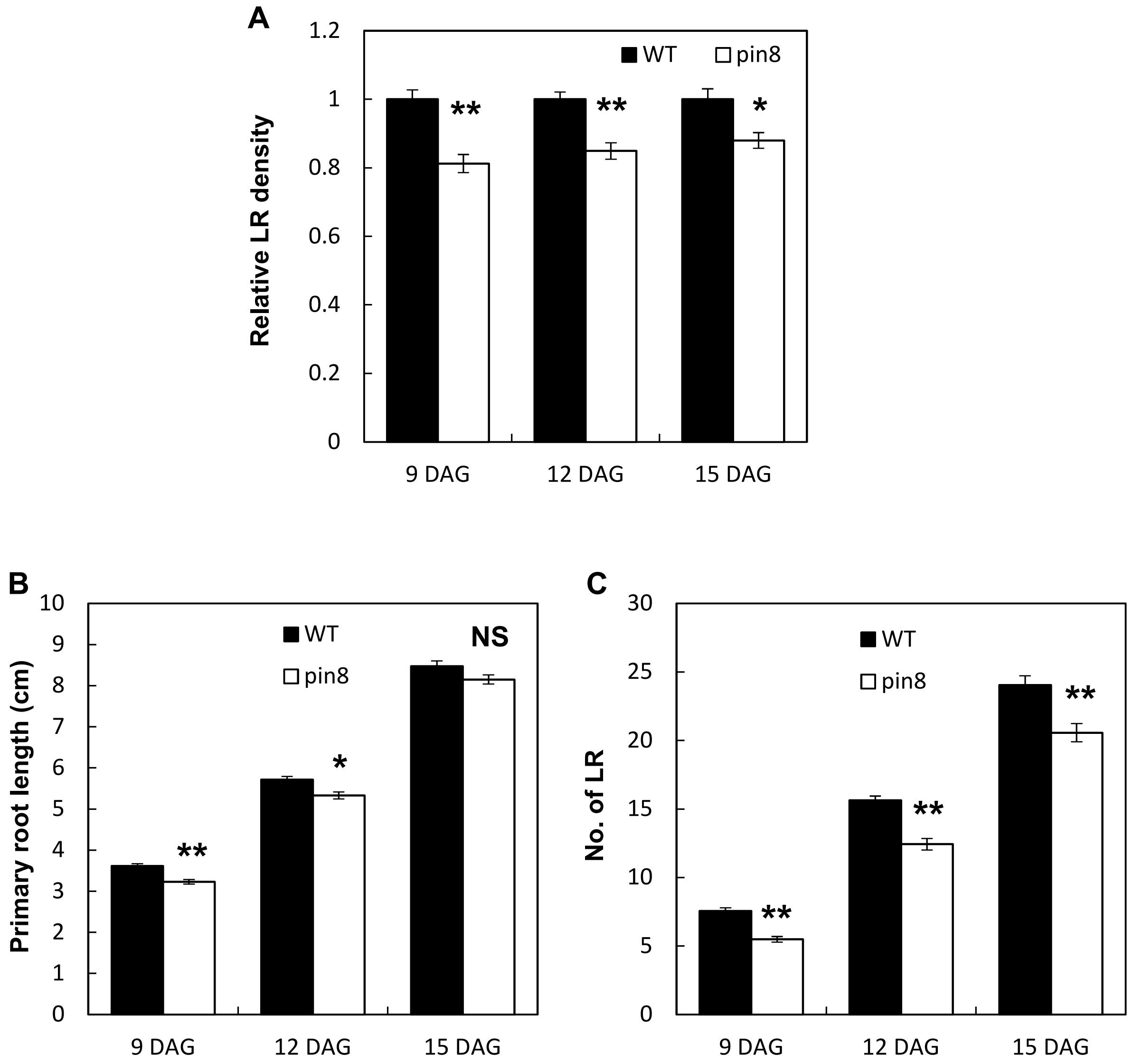

Figure S3. PIN8 affects lateral root (LR) development. **(A-D)** LR densities (number of LRs per cm of the root, [**A**]), primary root length **(B)**, and number of LR **(C)** of the wild type (WT) and *pin8* mutant from 9 to 15 days after germination (DAG). Data represent mean ± SE (n = 37–40 seedlings). Significant differences compared with WT are indicated using asterisks (*$P < 0.01$, **$P < 0.001$; Student's t-test).

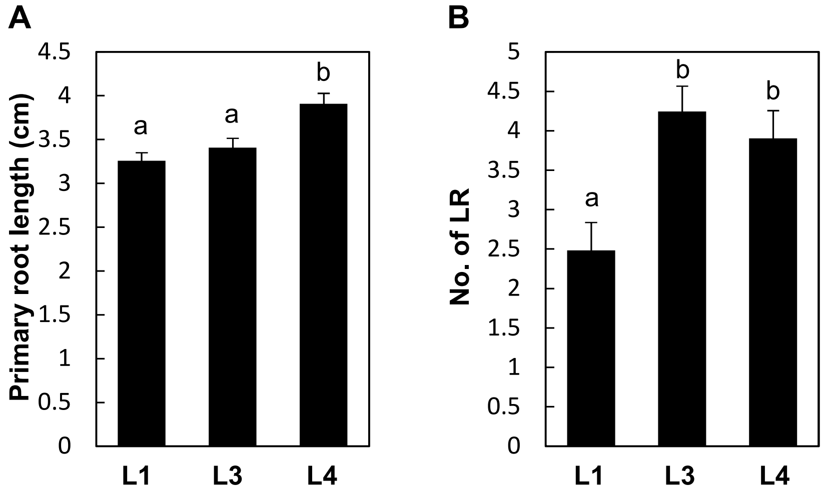

Figure S4. Primary root length **(A)** and number of LR **(B)** of three independent *ProPIN8:PIN8:GFP* transgenic lines. Data represent mean ± SE (n = 27–66 seedlings). Statistically significant differences were determined using one-way analysis of variance (ANOVA) with Tukey's unequal N HSD post hoc test and are denoted with different letters ($P < 0.05$).

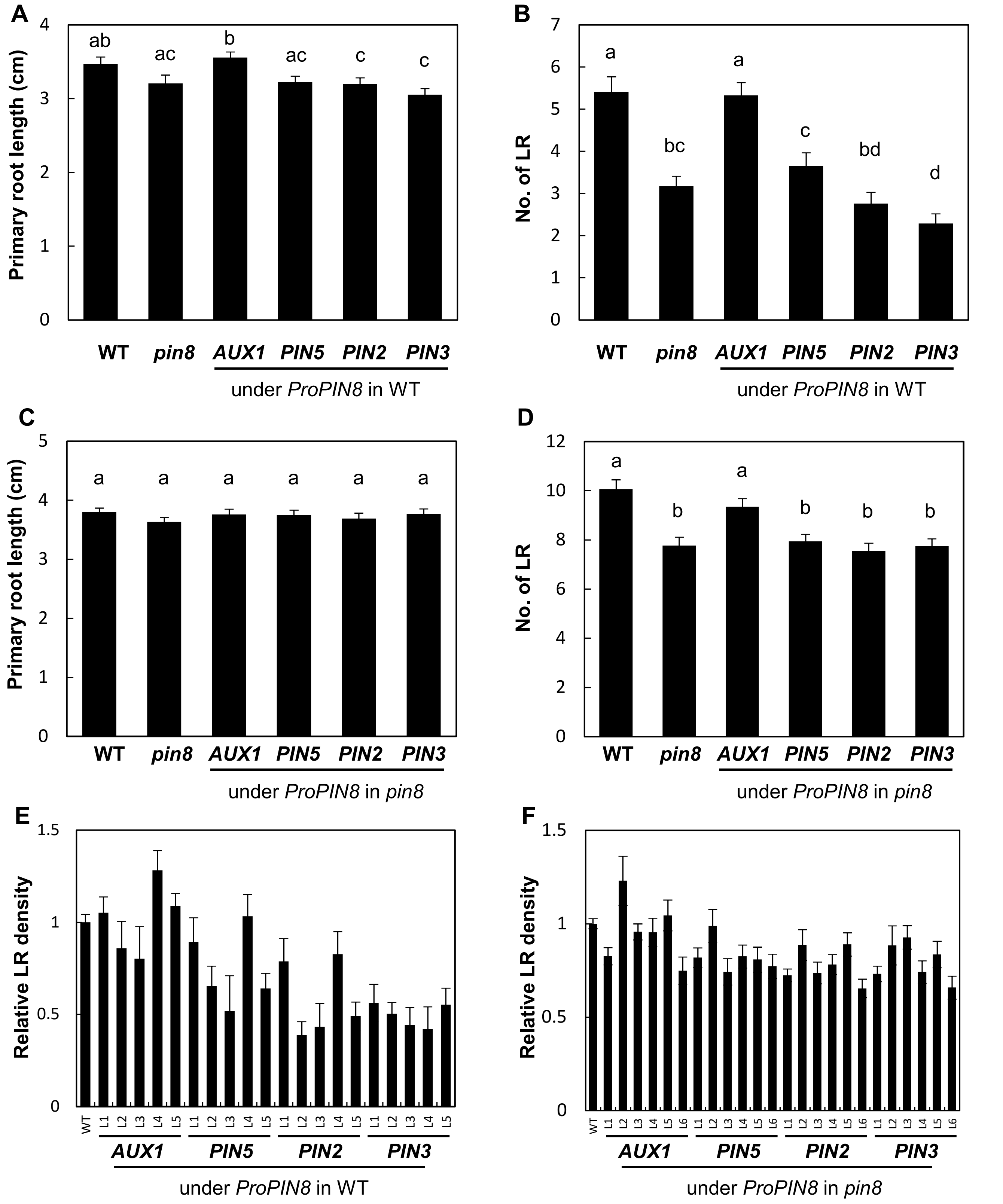

Figure S5. Primary root length and number of LR of the WT, *pin8* mutant, and transgenic lines expressing *ProPIN8*-driven auxin transporter genes in the WT (**A** and **B**) and *pin8* mutant (**C** and **D**) backgrounds, and relative LR density of independent lines for each transgenic construct (**E** and **F**). Data represent mean ± SE (n = 29–37 seedlings for **A** and **B** and 61–68 seedlings for **C** and **D**). Statistically significant differences are denoted with different letters ($P < 0.05$; one-way ANOVA with Tukey's unequal N HSD post hoc test).

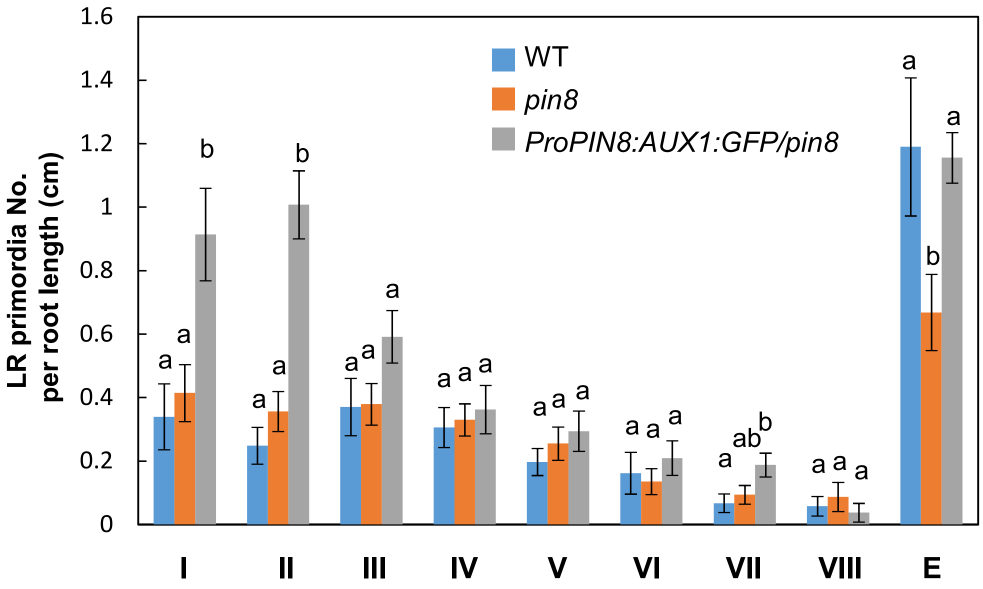

Figure S6. Distribution of LR primordia of the wild type (WT), *pin8* mutant, and *pin8*-complementation line expressing *ProPIN8:AUX1:GFP* at different developmental stages. Data represent mean ± SE (n = 17–20 seedlings). Statistically significant differences were determined using one-way analysis of variance (ANOVA) with Tukey's unequal N HSD post hoc test and are denoted with different letters ($P < 0.05$).

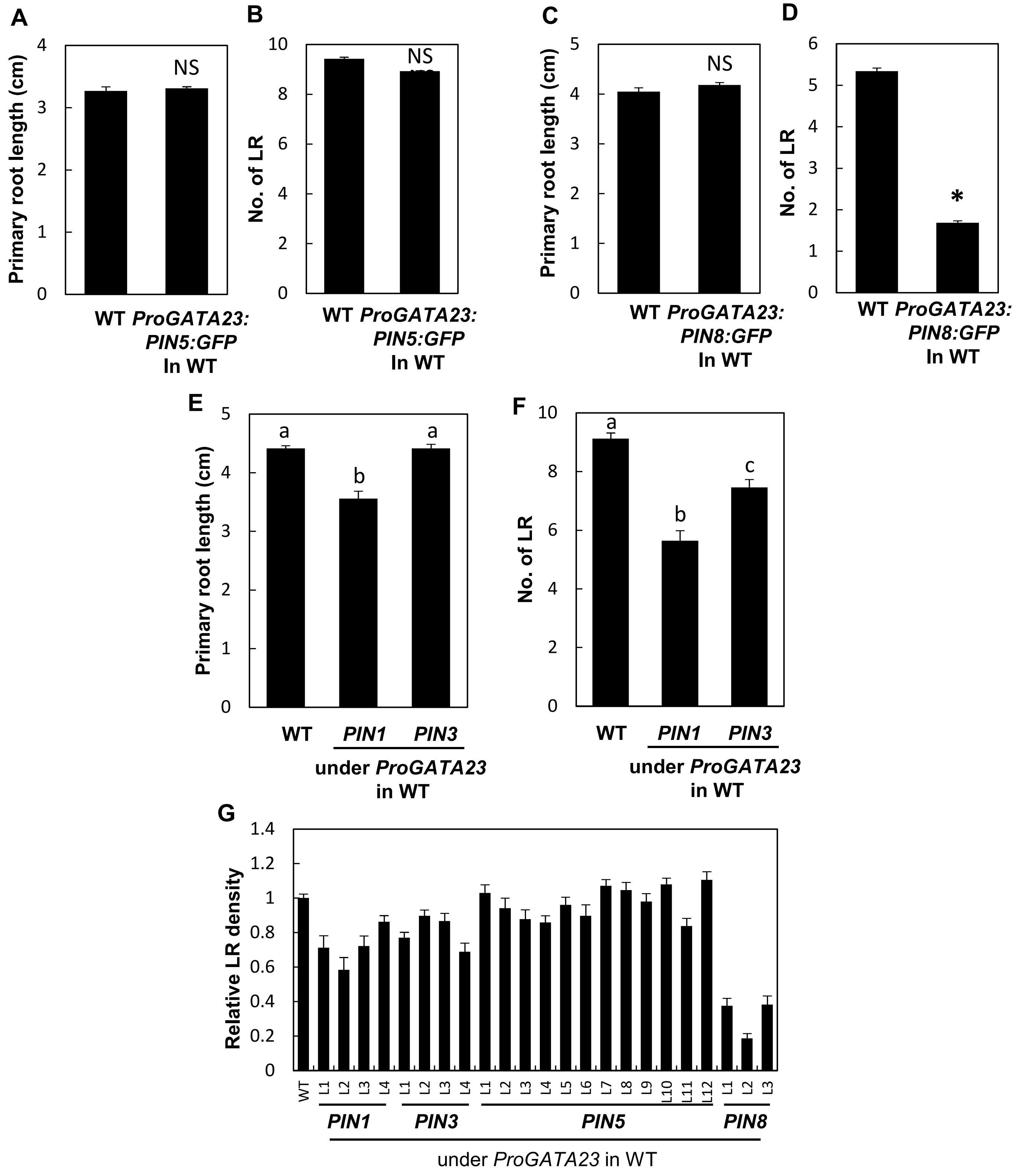

Figure S7. Primary root length, LR number, and relative LR density of the WT and *ProGATA23*-driven *PIN:GFP*-expressing transgenic lines. Data represent mean ± SE (n = 123–694 seedlings from 12 independent lines for *PIN5* [**A** and **B**], 106–231 seedlings from 3 independent lines for *PIN8* [**C** an **D**], and 89–120 seedlings from 4 independent lines for *PIN1* and *PIN3* [**E** and **F**]). (**G**) Relative LR density of independent transgenic lines for each construct. Significant differences compared with WT value are indicated using asterisks (*$P < 10^{-32}$; NS, not significant; Student's *t*-test [**A-D**]) and by different letters (P < 0.05; one-way ANOVA with Tukey's unequal N HSD post hoc test [**E** and **F**]).

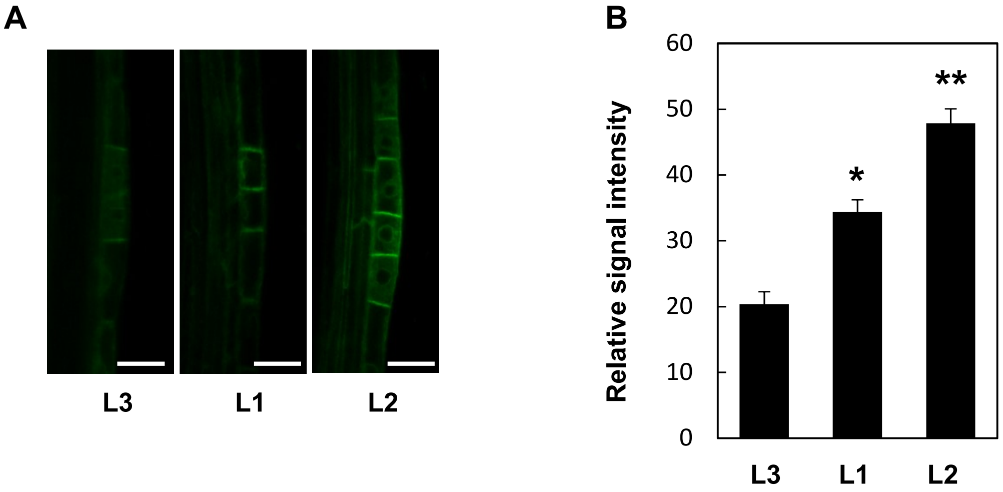

Figure S8. Localization of PIN8:GFP in the *GATA23* domain. **(A)** Confocal images of the PIN8:GFP signal in the root pericycle cells of three *ProGATA23:PIN8:GFP* transgenic lines with different PIN8:GFP expression levels. Scale bar = 20 μm. **(B)** Relative PIN8:GFP signal intensities of three *ProGATA23:PIN8:GFP* lines. Data represent mean ± SE (n = 6–9 seedlings). In **B**, significant differences compared with L3 intensity are indicated using asterisks (*$P < 10^{-3}$, **$P < 10^{-5}$; Student's t-test).

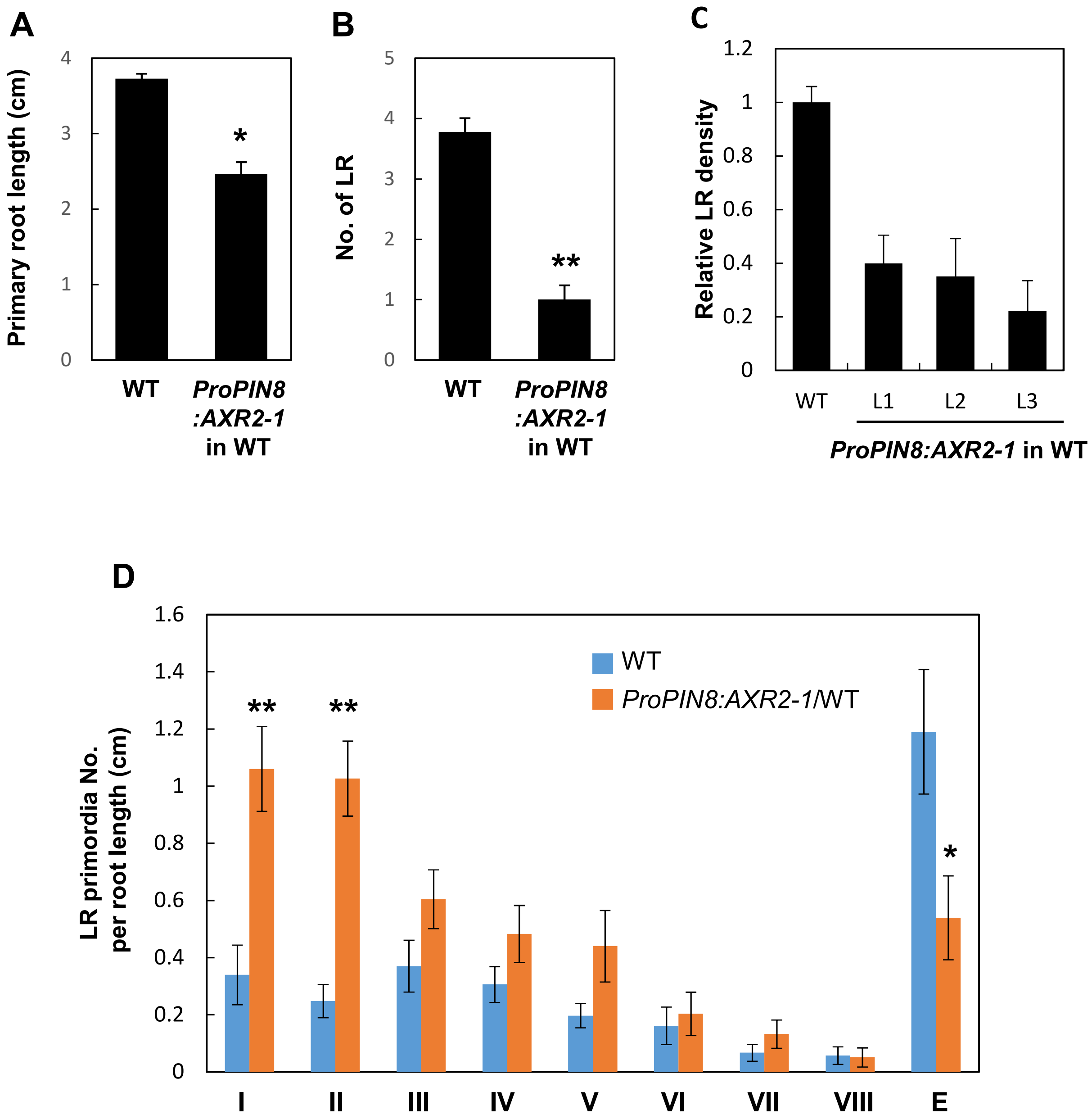

Figure S9. Auxin signaling in the *PIN8* domain is required for LR development. **(A)** Primary root length of the WT and *ProPIN8:AXR2-1:GFP* transgenic lines. **(B)** Number of LR of WT and *ProPIN8:AXR2-1:GFP* transgenic lines. (**C**) Relative LR density of independent lines of the *ProPIN8:AXR2-1:GFP* transformant. Data represent mean ± SE (n = 35–36), and significant differences compared with the WT are indicated using asterisks (*$P < 10^{-9}$; **$P < 10^{-11}$; Student's t-test [**A** and **B**]). (**D**) Distribution of LR primordia of WT and *ProPIN8:AXR2-1:GFP* transgenic lines at different developmental stages. Data represent mean ± SE (n = 15–17 seedlings). Significant differences compared with WT are indicated using asterisks (*$P < 0.01$, **$P < 0.001$; Student's t-test).

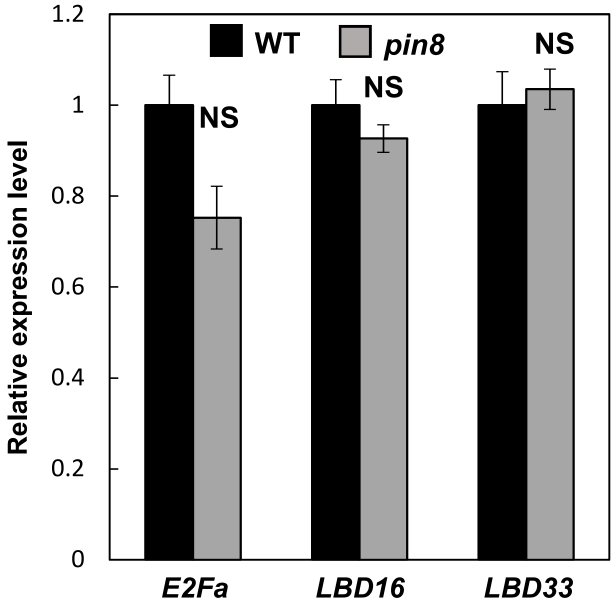

Figure S10. Effect of *PIN8* mutation on the expression of genes involved in lateral root (LR) development. Quantitative real time RT-PCR analysis of lateral root development genes in wild type (WT) and *pin8* mutant. Data are the mean ±SE of at least three independent biological replication. Differences are not significant (NS) from the WT transcript level in Student's *t*-test (*P*>0.1).

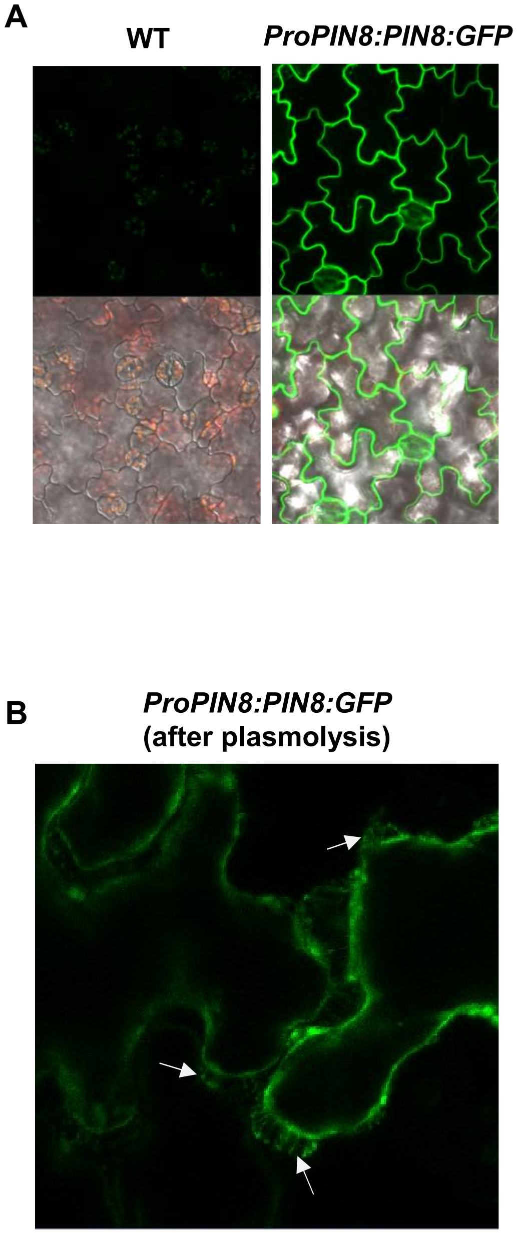

Figure S11. PIN8 localized to the PM in the cotyledon epidermal cells. (**A**) GFP signals in the cotyledon epidermal cells of 3-d-old WT and *ProPIN8:PIN8:GFP* transformant seedlings. Upper panel, dark field; lower panel, dark and bright field overlapped. (**B**) GFP signals from the cotyledon pavement cells of *ProPIN8:PIN8:GFP* transformant seedling after plasmolysis, suggesting the PM localization of PIN8 where PIN8-including PM is strongly associated with the cell wall as indicated by hectian strands (arrow).
